# A Portable Automated Microfluidic Platform for Point-of-Care Testing for Multiple Mycotoxins in Wine

**DOI:** 10.3390/foods13132066

**Published:** 2024-06-28

**Authors:** Jun Liu, Shiyu Zeng, Haoyu Zhu, Xinhua Wan, A. S. M. Muhtasim Fuad Sohan, Binfeng Yin

**Affiliations:** 1 Suqian Product Quality Supervision and Inspection Institute, Suqian 223800, China; ljljljlj1982@163.com; 2 School of Mechanical Engineering, Yangzhou University, Yangzhou 225127, China; shiyuzeng315@foxmail.com (S.Z.); zhuhaoyulw@163.com (H.Z.); usawwns@163.com (X.W.); 3 School of Electrical and Mechanical Engineering, The University of Adelaide, Adelaide, SA 5005, Australia; asmmuhtasimfuad.sohan@student.adelaide.edu.au

**Keywords:** mycotoxins, point-of-care testing (POCT), portable automated microfluidic platform (PAMP), chemiluminescence (CL)

## Abstract

Food safety requires point-of-care testing (POCT) for mycotoxins, since their presence in wine significantly impacts the wine industry and poses a severe threat to human life. Traditional detection methods are usually limited to detecting one mycotoxin and cannot achieve high-throughput, automated, and rapid quantitative analysis of multiple mycotoxins in real samples. Here, we propose a portable automated microfluidic platform (PAMP) integrating a chemiluminescence (CL) imaging system and a microfluidic chip to realize POCT for multiple mycotoxins in real samples, simplifying complex manual operations, shortening the detection time, and improving the detection sensitivity. Specially, silicone films were used as substrates on microfluidic chips to incubate mycotoxin conjugations, and the streptavidin–biotin (SA-B) system and an indirect immunoassay were implemented on silicone films to improve the sensitivity of reaction results. Interestingly, these methods significantly improved detection results, resulting in sensitive detection of mycotoxins, including zearalenone (ZEA) ranging from 1 to 32 ng/mL, aflatoxin B1 (AFB1) ranging from 0.2 to 6.4 ng/mL, and ochratoxin A (OTA) ranging from 2 to 64 ng/mL. The recovery of samples reached 91.39–109.14%, which verified the reliability and practicability of the PAMP. This PAMP enables sensitive and rapid detection of multiple mycotoxins in markets or wineries that lack advanced laboratory facilities. Therefore, it is essential to develop a portable microfluidic platform for POCT to detect mycotoxins in real samples.

## 1. Introduction

Mycotoxins [1] are secondary metabolites of mold [2], presenting severe reproductive toxicity [3], teratogenicity [4], and carcinogenicity [5]. Mycotoxins such as aflatoxin B1 (AFB1) [6], ochratoxin A (OTA) [7], and zearalenone (ZEA) [8] usually contaminate wine through raw materials [9,10,11,12]. The additive, synergistic, or antagonistic effects of mycotoxins enhance their harm to human health and drive a demand for the classification of multiple mycotoxins [13,14,15]. Mycotoxins’ high toxicity [16] has led many countries and organizations to set strict standards for mycotoxins in wine at the ppb level [9,17]. Given the mass consumption of wine, developing a sensitive, low-cost, and rapid detection method for multiple mycotoxins is urgent [18,19,20,21,22].

Most countries and organizations advocate high-performance liquid chromatography (HPLC) [23,24] and liquid chromatography–mass spectrometry (LC-MS) [25] as standard detection methods for mycotoxins. Although these conventional analysis methods are sensitive and accurate, they are limited in practical applications due to time-consuming procedures, high cost, dependence on instruments, and requirements for professional skills [26,27]. Researchers have reported diverse biosensors for mycotoxin analysis [28]. Pagkali et al. developed an immunosensor based on a single-chip Mach–Zehnder interferometer (MZI) array integrated into a silicone chip that allows rapid, labeling-free determination of AFB1, fumonisin B1 (FB1), and deoxynivalenol (DON) in beer samples [29]. Zhu et al. developed a colorimetric biosensor for dual mycotoxin detection, achieving detection ranges of 5–250 ng/mL for AFB1 and 0.5–80 ng/mL for OTA, respectively [30]. Zheng et al. reported a multiplex immunochromatographic assay (ICA) that uses functionalized magnetic quantum dots (QDs) as a capture/detection bi-functional immune probe to simultaneously and sensitively detect AFB1, OTA, and FB1 in food with minimum detection limits of 0.42, 11.48, and 4.21 pg/mL [31].

Bioanalysis [32,33] benefits from the reduced reagent consumption, shortened reaction times, and improved user-friendliness conferred by microfluidic devices [34]. Many approaches have developed a variety of portable devices based on microfluidics for mycotoxin detection to meet the needs of on-site detection [35,36,37]. Lin et al. used a two-channel indium tin oxide (ITO) microfluidic electrochemical immunosensor to successfully detect FB1 and DON simultaneously [38]. Soares et al. reported a microfluidic competitive immune sensor based on multiple microspheres combined with an a-Si:H thin-film photodiode array for integrated fluorescence signal acquisition to achieve ultra-fast AFB1, OTA, and DON detection [39]. However, sample pretreatment and signal acquisition need additional laboratory instruments.

Microfluidic chips, which can be operated manually or automated to a high degree, offer an effective solution for the real-time detection of multiple mycotoxins. Their sensitivity, detection speed, and cost advantages make them widely used in food safety, medical diagnostics, and other detection fields. By integrating multiple detection methods, such as chemiluminescence [40], fluorescence [41], colorimetry [42], and immunoassays [43], suitable microfluidic chips can be fabricated for the detection of most biomarkers. However, many microfluidic chips still require some human operation and do not support fully automated point-of-care testing (POCT) for multiple biomarkers. Therefore, developing a portable automatic microfluidic platform is essential.

**Scheme 1:**
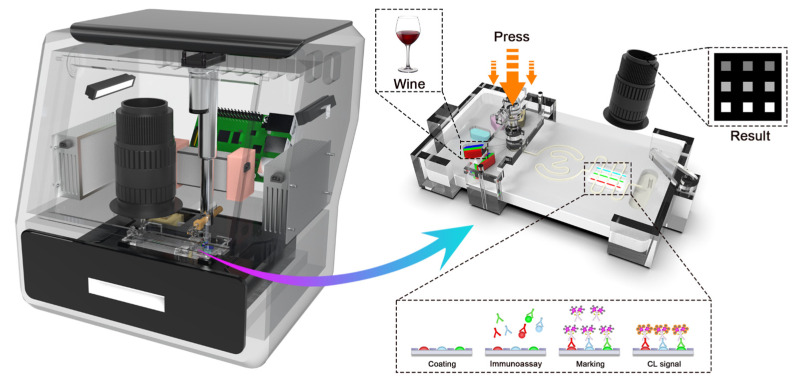
Scheme of the design and application of the PAMP for detecting mycotoxins.

Herein, we proposed a microfluidic chip integrating multiplexed detection, a micromixer, chemiluminescence, and the streptavidin–biotin (SA-B) system to implement POCT for OTA, ZEA, and AFB1. In the microfluidic chip, an indirect competitive immunoassay was integrated into the reaction layer made of silicone film. A circular-based, centrosymmetric micromixer achieved sensitive detection of mycotoxins. Subsequently, a portable automated microfluidic platform (PAMP) was established to control the flow and testing of the sample, reducing the complexity of manual experiments and significantly improving the flexibility and sensitivity of experiments while reducing the detection time. In the PAMP, based on an indirect competitive immunoassay, OTA, ZEA, and AFB1 in wine can be detected using the immunoreaction on the reaction layer (Scheme 1). The PAMP meets the requirements of POCT and will be particularly applicable for monitoring multiple mycotoxins in wine without advanced laboratory facilities.

## 2. Materials and Methods

### 2.1. Materials and Instruments

Various materials and chemicals were utilized in this study to manufacture the microfluidic chip for the PAMP. Specifically, we prepared PDMS, adding Sylgard 184 purchased from Dow Corning Inc. (Midland, MI, USA) and silicone films (ST-44) purchased from Shanghai Shentong Rubber and Plastic Products Co., Ltd. (Shanghai, China). The detecting reagents, including OTA–ovalbumin (OTA-OVA), ZEA–ovalbumin (ZEA-OVA), and AFB1–ovalbumin (AFB1-OVA) conjugates; antibodies against OTA, ZEA, and AFB1; and OTA, ZEA, and AFB1 standards, were purchased from Zhejiang Zhunce Biotechnology Co., Ltd. (Zhejiang, China). Then, a detection antibody conjugated with biotin (B-IgG) and HRP conjugated with streptavidin (SA-HRP) were acquired from Thermo Fisher Scientific (Waltham, MA, USA). BSA fraction V powders were purchased from Tianjin Kangyuan Biotechnology Co., Ltd. (Tianjin, China). PBS tablets and Tween-20 were bought from Amresco (Solon, OH, USA). Supersensitive chemiluminescent substrate kits were obtained from Beijing Labgic Technology Co., Ltd. (Beijing, China). Other common reagents were of analytical grade and were provided by Macklin Inc. (Shanghai, China). PBST was allocated by adding 0.5% Tween-20 to phosphate buffer.

We fabricated the PDMS chip using a YXIN-PRO 3D printer from Yangzhou YIXIN 3D Technology Co., LTD (Yangzhou, China). The bonding process for the microfluidic chip was achieved with a PTL-VM500 plasma cleaner from Putler (Suzhou, China). Photographs of the microchannels were taken with a TM28 metallographic microscope from Shenzhen AOSVI (Shenzhen, China). The controlled inflow of distinct sample solutoions was managed using the ISPLab02 intelligent syringe pump from DK InfuseTek (Shanghai, China). Chemiluminescence signals were captured using a QHY22 CCD camera from Light Speed Vision Co., LTD. (Beijing, China).

### 2.2. Design of the Microfluidic Chip

The design of a microfluidic chip contains four parts: the PDMS layer, the reaction layer, the pressure valve, and the bracket. First, we used SolidWorks^®^ 2016 software to design the top and bottom layers of the PDMS layer. The upper layer, 68 mm long, 35 mm wide, and 4 mm thick, contained five capsule-type liquid reservoirs 7 mm long, 3 mm wide, and 3 mm thick, which could store 57 μL of reagents. The total length of the microchannel in the upper layer, with a cross-section size of 0.4 mm × 0.4 mm, was 187.38 mm. Six vents were drilled at the end of each reservoir and microchannel to introduce the reagent and balance the pressure. A 5 mm diameter valve hole and a pressure valve were designed to reasonably control the flow of reagents in the five reservoirs. To avoid leakage of the pressure valve, the diameter of the insert part of the pressure valve was set to 5.2 mm to create an interference fit with the valve hole. We also designed a circular centrosymmetric micromixer for efficient binding between antigens and antibodies. Subsequently, a wave-shaped microchannel attached to the end of the micromixer was designed for mycotoxin detection. The lower PDMS layer was the same size as the upper layer, except that the lower layer did not contain any microchannel structure, and there was a waste liquid pool 17 mm long, 5 mm wide, and 3 mm thick at the end to store the reagent after the reaction. The reaction layer was a 10 mm long, 10 mm wide, 0.3 mm thick silicone film coated with different antigens between double layers of PDMS. The bracket design specifically involved multiple parts for support at both ends and the middle of the chip. The brackets at both ends of the chip limited the movement of the chip, and the brackets in the middle assisted in the positioning of the pressure valve to reduce the leakage phenomenon caused by instability of pressure.

### 2.3. Fabrication of the Microfluidic Chip

**Figure 1:**
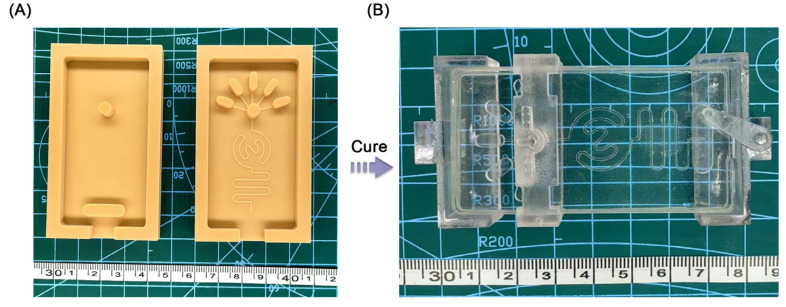
(**A**) The microfluidic molds manufactured by a DLP 3D printer. (**B**) The cured microfluidic chip, printed valve, and bracket.

**Figure 2:**
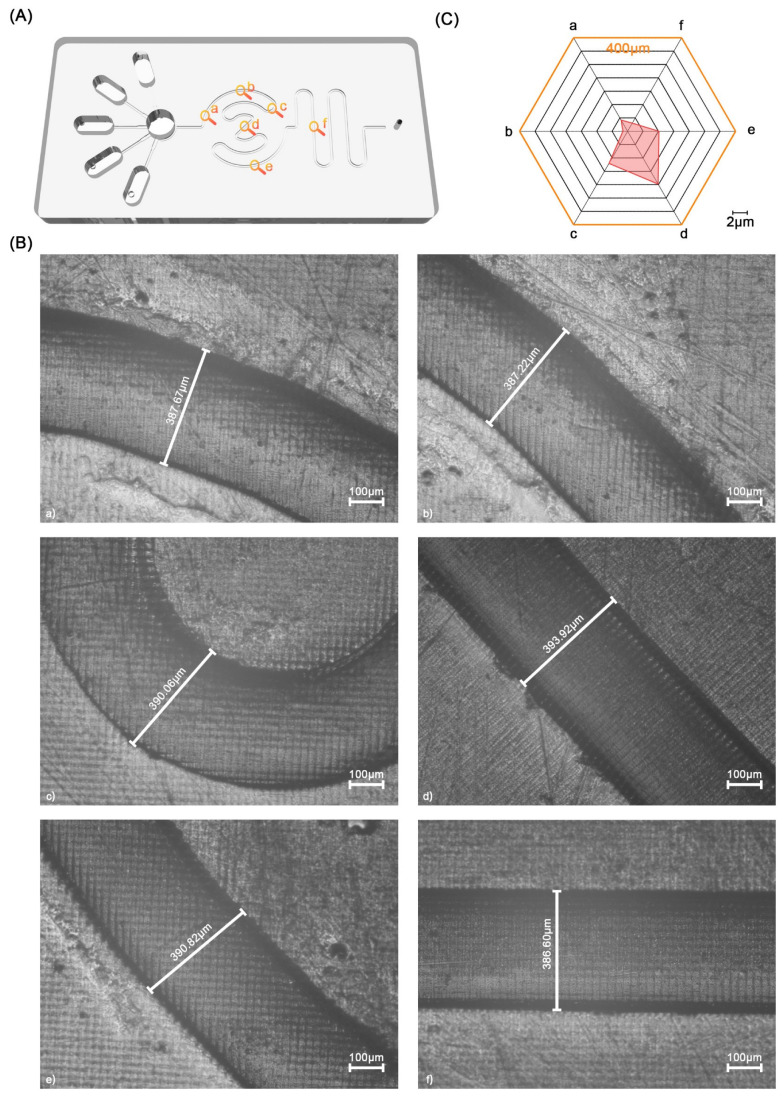
(**A**) The locations of the proposed interception. (**B**) Photographs of the six locations (**a**–**f**) of microchannel with the size ranging from 386.60 μm to 393.92 μm. (**C**) Quantitative analysis of the microchannel; (**a**–**f**): corresponding dimensions of six locations in the microchannel.

To put the designed microfluidic chip into practice, the double layer of PDMS started with molds (Figure 1) manufactured using a DLP 3D printer [44,45]. After surface treatment of the mold, a mixture containing PDMS and curing agent was prepared in a ratio of 10:1 [46]. After degassing, the mixture was introduced into a vacuum oven to eliminate air bubbles and poured into a treated mold. Despite the mold treatment, surface bubbles sometimes still appeared during the pouring stage. These undesirable bubbles were effectively eliminated by applying airflow to expel the trapped air. The mold containing the PDMS mixture was then subjected to a three-hour curing process in a dedicated oven at a temperature of 65 °C. After the curing process, the microchannels and base microchannels were carefully separated from the mold using tweezers and then cleaned to remove debris. The five reservoirs were also established on the chip by punching holes 1 mm in diameter. After pretreating the double PDMS layers of the microfluidic chip using a plasma cleaner configured with parameters including a power output of 200 W and an oxygen flow rate of 1.5 L/min, the silicone films coated with multiple antigens were bonded together, with a bonding period of 60 s. Furthermore, the local microchannels in microfluidic chips were captured through a metallographic microscope to evaluate the accuracy of the fabrication process for the microfluidic chips. To evaluate the printing accuracy of microchannels, we observed and measured the selected channels (a–f) through the metallographic microscope as shown in Figure 2. Compared with the design size of 400 μm, the measured channels exhibited accuracy with an error range of 1.52–3.35%, indicating the excellent processing accuracy of the microfluidic chips.

### 2.4. Lab on a Microfluidic Chip

To put the fabricated microfluidic chip into practical application, we propose a novel approach to quantifying the concentration of mycotoxins in complex environments. First, in addition to the liquid to be tested, we injected 30 μL of different detection reagents, including capture antibody, PBST, detection antibody, and chemiluminescent substrate, into four liquid reservoirs. Next, we introduced 30 μL of the agent to be tested into the corresponding liquid reservoir. The pores at the end of the microfluidic chip were then connected to a negative-pressure pump through a silicone tube to drive the flow of the reagent under negative pressure. Next, multi-channel pressure valves controlled the fluid flow from the five reservoirs. Initially, the pressure valve was fully closed to seal the liquid in the five reservoirs completely. When pressing down the valve to the first stage, the capture antibody and the agent to be tested in the first and second liquid storage tanks were pumped into subsequent micromixers for mixing and finally stayed in the wavy microchannels on the reaction layer for incubation. After incubation, the pressure valve was pressed down to the second stage, and half of the PBST in the third reservoir was pumped out to clean the reaction layer. The pressure valve was continuously pressed down to the third stage; at this point, the detection antibody in the fourth reservoir was pumped out and then remained stationary in the wavy-shaped microchannel for incubation. After the second incubation, the pressure valve was raised to the second stage to allow the remaining PBST to be pumped out to clean the reaction layer. Finally, the pressure valve was pressed down to the fourth stage. The chemiluminescent substrate was pumped out, producing a chemiluminescent signal on the reaction layer that the CCD camera could observe. The observed chemiluminescence signal has a linear relationship with the concentration of the liquid to be measured.

### 2.5. Imaging Detection

The indirect immunoassay was used to detect ZEA, AFB1, and OTA concentrations in wine samples, with chemiluminescence (CL) serving as the readout signal. The microfluidic chip was then placed in the CCD camera system. After injecting the CL substrate into the chip, CL imaging and intensity were recorded with GEL-PRO Analyzer 4.0, capturing the photo at an exposure time of 5 s.

### 2.6. Statistical Evaluation

To evaluate the effectiveness of PAMP detection, the limit of detection (LOD), recovery, and corresponding relative standard deviation (RSD) were calculated to compare with those of other detection methods. We used the formula of three times the signal-to-noise ratio to calculate the LOD:

(1) $$\begin{array}{c} LOD=\frac{3\sigma }{k} \end{array}\;$$

where σ is the standard deviation of the five sets of blank values and k is the slope of the standard curve.

Recovery was calculated by following this formula:

(2) $$\begin{array}{c} Recovery=\left(\frac{Q_{ds}-Q_{bs}}{Q_{s}}\right)\times 100\% \end{array}$$

where Q_s_ is the quantity of the spiked sample, Q_bs_ is the quantity of basic sample, and Q_ds_ is the detected quantity of the spiked sample.

The corresponding RSD was calculated following this formula:

(3) $$\begin{array}{c} RSD=\frac{\sqrt{\frac{\sum_{i=1}^{n}{\left(x_{i}-\bar{x}\right)}^{2}}{n-1}}}{\bar{x}}\times 100\% \end{array}$$

where $x_{i}$ is the calculated recovery and $\bar{x}$ the average of the recovery.

## 3. Results and Discussion

### 3.1. The Establishment of PAMP

**Figure 3:**
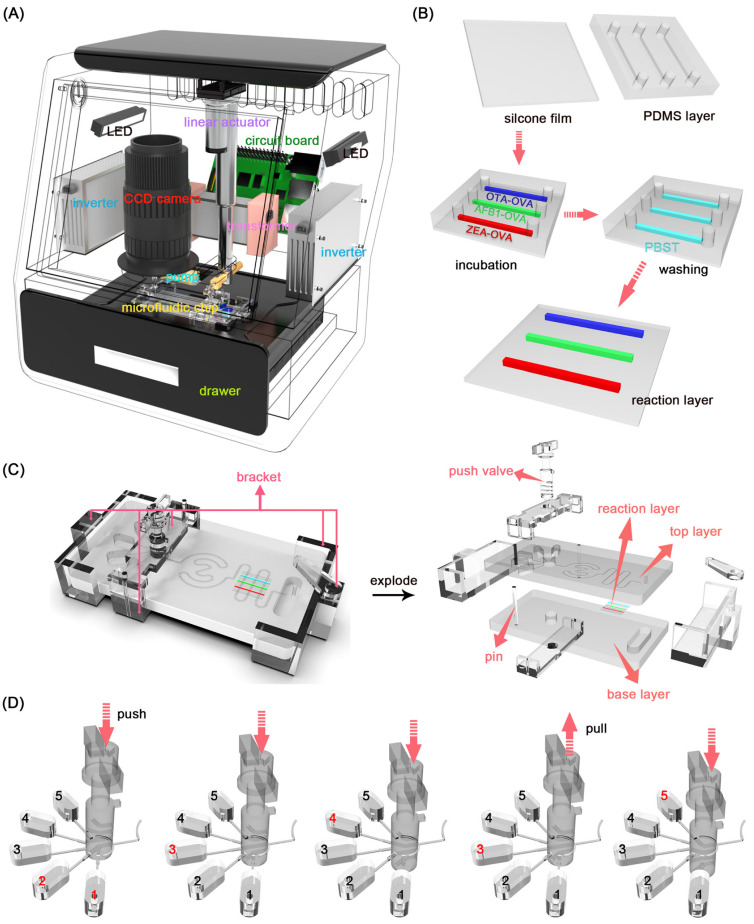
(**A**) Characterization of the PAMP. (**B**) The procedures for applying antigen coatings and blocking the silicone film. (**C**) Exploded and assembled drawings of the microfluidic chip. (**D**) Microvalve manipulation states for quantitative analysis. 1–5: Five reservoirs of the microfluidic chip storing sample, capture antibody, PBST, IgG-B-SA-HRP, and CL substrate, respectively.

In order to achieve automated detection of the target, a PAMP was constructed, greatly simplifying the immunization procedure and shortening the detection time. As shown in Figure 3A, the PAMP consisted of two layers. The upper layer mainly contained a CCD camera, inverters, transformers, a linear actuator, circuit boards, and LED lights. Specifically, the CCD camera was mounted at a specific location with a through hole for real-time observation of the chemiluminescence signals within the chip, while the linear actuator was suspended to control the microfluidic chip. The lower layer mainly contained a drawer, a microfluidic chip, and a negative-pressure pump. The negative-pressure pump and microfluidic chip were assembled in the drawer and pushed into the PAMP. The cured PDMS layer was placed on a silicone film to prepare a reaction layer for detecting multiple mycotoxins. Three microchannels on the PDMS layer were injected with three different antigens for coating. After incubation at 37 °C for 15 min, the excess antigen was washed away with PBST. The reaction layer was prepared after removing the PDMS layer (Figure 3B). As shown Figure 3C, the microfluidic chip located in the lower layer of the PAMP was mainly divided into the top layer, the reaction layer, the base layer, and a pressure valve. Specifically, the top layer mainly contained five capsule-type reservoirs, microchannels for mixing and reaction, and an air hole connected to the negative-pressure pump. The reaction layer was a silicone film coated with different antigens. The base layer had a waste liquid pool, which stored waste liquid and seals microchannels. Subsequently, to realize the precise control of the microfluidic chip and avoid corrosion of the instrument caused by direct contact, we designed a set of brackets with the chip, which could prop up the chip and reasonably limit the pressure valve. When the microfluidic chip equipped with the bracket was pushed into the PAMP, the electric push rod equipped with the connecting rod was connected to the press valve to drive the lifting and lowering of the press valve. As shown in Figure 3D, to realize the precise control of the liquid in the five liquid storage pools by the microvalve, the electric push rod controlled the microvalve with different channels in the four layers to communicate with the microchannel. First, to avoid liquid leakage from the reservoir, the microvalve was located in the highest position to ensure air tightness. Then, when the microvalve is pressed down until the first layer channel was connected with the microchannel, the sample in the reservoir 1 and the capture antibody in the reservoir 2 flowed along the microchannel. Later, PBST, IgG-B-SA-HRP, and CL substrate in the corresponding reservoirs 3, 4, and 5 flowed along the microchannel when the microvalve descended to the second, third, and fourth layers. In addition, due to the flexibility of the connecting rod, half of the liquid in the third reservoir flowed out along the channel at the second step, as shown in Figure 3D. The remaining half of the liquid flowed out along the channel in the fourth step by lifting the connecting rod to ensure the integrity and accuracy of the immune response.

### 3.2. The Mixing Performance of Micromixer

**Figure 4:**
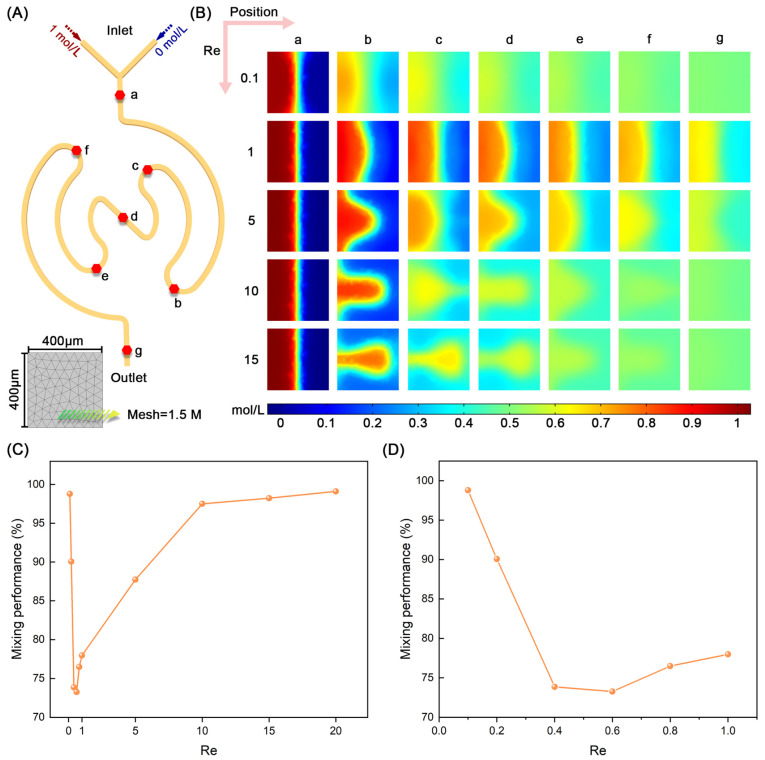
(**A**) Schematic diagram of the micromixer. (**B**) The mixing performance of each cross-section at different Re values. (a–g): Six locations of the micromixer. (**C**) The mixing efficiency of the microchannel exit at Re values ranging from 0.1 to 20. (**D**) The mixing efficiency of the microchannel exit at Re values ranging from 0.1 to 1.

Since the mixing efficiency in microfluidic chips will affect the binding efficiency of antibodies and antigens in microfluidic chips and the sensitivity of chemiluminescence detection, it is very significant to study the mixing efficiency of microchannels. In order to evaluate the mixing efficiency of the designed mixer, channel length and Reynolds number were used as the main study variables. As shown in Figure 4B, when the Reynolds number increases from 1 to 15, the concentration dividing line of the remaining cross-section becomes curved and fuzzy except for cross-section A, which indicates that the mixing efficiency gradually increases. When Re = 10 and 15, the mixing efficiency at the end of the microchannel in Figure 4C exceeds 95%, which means an excellent mixing effect. However, when Re = 0.1, the mixing efficiency at its exit is close to that at the Reynolds number of 10 or 15, which is different from the mixing efficiency at Re = 1, indicating that there is a turning point in the mixing efficiency when the Reynolds number changes from 0.1 to 1. As shown in Figure 4D, when Re = 0.1, the liquid in the mixer mainly relies on intermolecular diffusion for mixing to achieve higher mixing efficiency due to its slow flow rate. When Re changes from 0.1 to 1, the mixing efficiency of the outlet decreases significantly at first until the mixing efficiency becomes gradually stable when Re is in the range of 0.4–0.6. When Re is more significant than 0.6, the flow rate will be the main factor affecting the mixing efficiency. However, the long flow time at Re = 0.1 cannot meet the need for rapid and sensitive detection of multiple mycotoxins. In addition, with the increase of Re, the higher negative pressure of the driving fluid is more likely to break the microchannel. Therefore, we chose Re = 10 as the flow parameter of the microfluidic chip, which ensures high mixing efficiency and controllability.

### 3.3. The Microfluidic Chip for Detection

**Figure 5:**
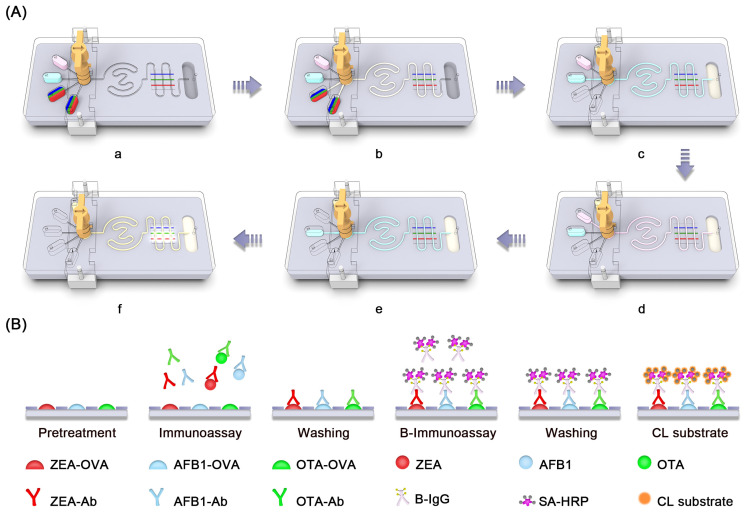
(**A**) Scheme of the immunoassay in the microfluidic chip. The detection steps include injection (**a**), competitive immunoassay (**b**,**d**), cleaning (**c**,**e**), and CL stimulation (**f**). (**B**) The detection principle for detecting ZEA, AFB1, and OTA.

Mycotoxins, including ZEA, AFB1, and OTA, are small molecules. Therefore, capturing antigens based on the traditional sandwich ELISA method is complex, and recording CL signals using a detection antibody combined with HRP is difficult. In contrast, competitive ELISA is suitable for detecting small molecules in complex sample mixtures. In contrast to direct immunoassay, the signal of indirect immunoassay is negatively correlated with the concentration of mycotoxins in the sample, which makes the detection signal weak and difficult to observe at the high concentration of mycotoxins. Therefore, we used a stable biotin-labeled detection antibody (IgG-B-SA-HRP) to improve the specificity of the immune response. In Figure 5, we used indirect competitive immunoassay to detect three mycotoxins: ZEA, AFB1, and OTA. The wine was injected through a needle into the reservoir. Then, the protocol for multiplex detection of ZEA, AFB1, and OTA by microfluidic chips began to immobilize conjugates of ZEA-OVA, AFB1-OVA, and OTA-OVA on the surface of the reaction layer on three parallel bands, respectively (Figure 5B). The silicone film was then sealed at the top and base layer of the PDMS, and BSA (5%) was injected into the chip to block the vacancies in the chip and the reaction layer. The sample and the capture antibody were then injected into the microchannel by the microvalve at Re = 10 for efficient mixing. The liquid was stationary when reaching the reaction layer for incubation. The targets in the mixture competed with the conjugate immobilized on the silicone membrane for the binding site of the capture antibody. After the immune reaction, the capture antibody bound to the target in the sample was washed away with PBST. Instead, the capture antibody that binds to the conjugates was attached to the silicone film. Subsequently, IgG-B-SA-HRP prepared by mixing SA-HRP with B-IgG was injected with Re = 10 into the microchannel and bound to the capture antibody attached to the silicone film. PBST washed away the excess capture antibodies. Finally, the chemiluminescent substrate was introduced into the microchannel and reacts with HRP to produce chemiluminescent signals. The high affinity between biotin and SA improves the specificity of the immune response. The above on-chip assays can be performed using a portable control system (Figure 3A), which simplifies the immunoassay process, helps to reduce assay time, and completes high-throughput assays (Figure 5A). Interestingly, the PAMP aims to fully automate the process from sample processing to detection, thereby simplifying operations and significantly enhancing detection accuracy. This provides essential support for in situ detection in domestic POCT applications.

### 3.4. Optimization of Coating Antigens and Capture Antibodies

**Figure 6:**
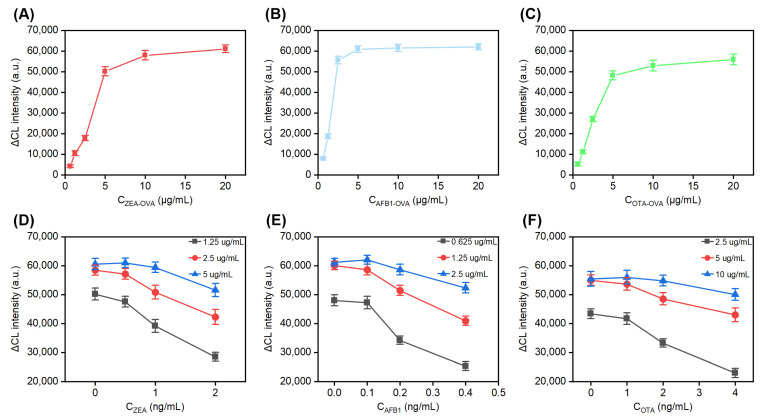
Concentration optimization of (**A**) ZEA-OVA, (**B**) AFB1-OVA, and (**C**) OTA-OVA. Concentration optimization of (**D**) ZEA-Ab, (**E**) AFB1-Ab, and (**F**) OTA-Ab.

We coated antigens with different concentrations to optimize concentrations of antigens to capture ZEA-Ab, AFB1-Ab, and OTA-Ab at the detection layer. We investigated ZEA-OVA, AFB1-OVA, and OTA-OVA of 0.625, 1.25, 2.5, 5, 10, 20 μg/mL for coating (Figure 6A–C). Our optimization condition was no standard substance; the concentration of Ab1 (ZEA-Ab, AFB1-Ab, and OTA-Ab) was 20 μg/mL, and IgG-B-SA-HRP was diluted at 1:1000. The other experimental conditions were consistent. Initially, CL intensity is positively correlated with the concentration of antigens. When the concentrations of antigens rise to a certain extent, the increase in CL intensity becomes inconspicuous. The optimal conditions of ZEA-OVA, AFB1-OVA, and OTA-OVA are 5, 2.5, and 5 μg/mL. To explore the optimal concentration of capture antibodies, we used the optimized antigen concentration as a condition, and Ab2 was diluted at 1:1000. The other experimental conditions are consistent. We set three concentrations of 1.25, 2.5, and 5 μg/mL as the optimization parameters of ZEA-Ab (Figure 6D). The results showed that the CL intensity of ZEA-Ab at 5 μg/mL is not much different from the blank group without mycotoxins, while the ZEA concentration was in the range of 0–1 ng/mL, so ZEA in the range of 0–1 ng/mL cannot be detected. When the concentration of ZEA-AB was 1.25 ng/mL, the chemiluminescence (CL) signal was significantly weaker compared to the other two groups. As the concentration of ZEA slightly increased, the CL signal further attenuated, making it difficult for a concentration of 1.25 ng/mL ZEA-AB to detect ZEA across a more comprehensive concentration range. In contrast, 2.5 μg/mL ZEA-Ab only does not detect 0–0.5 ng/mL of ZEA, which has a better detection limit and range. In summary, 2.5 μg/mL of ZEA-Ab was selected as the optimal concentration. Similarly, we chose 1.25 μg/mL AFB1-Ab and 5 μg/mL OTA-Ab as optimal concentrations.

### 3.5. Detecting Performance

**Figure 7:**
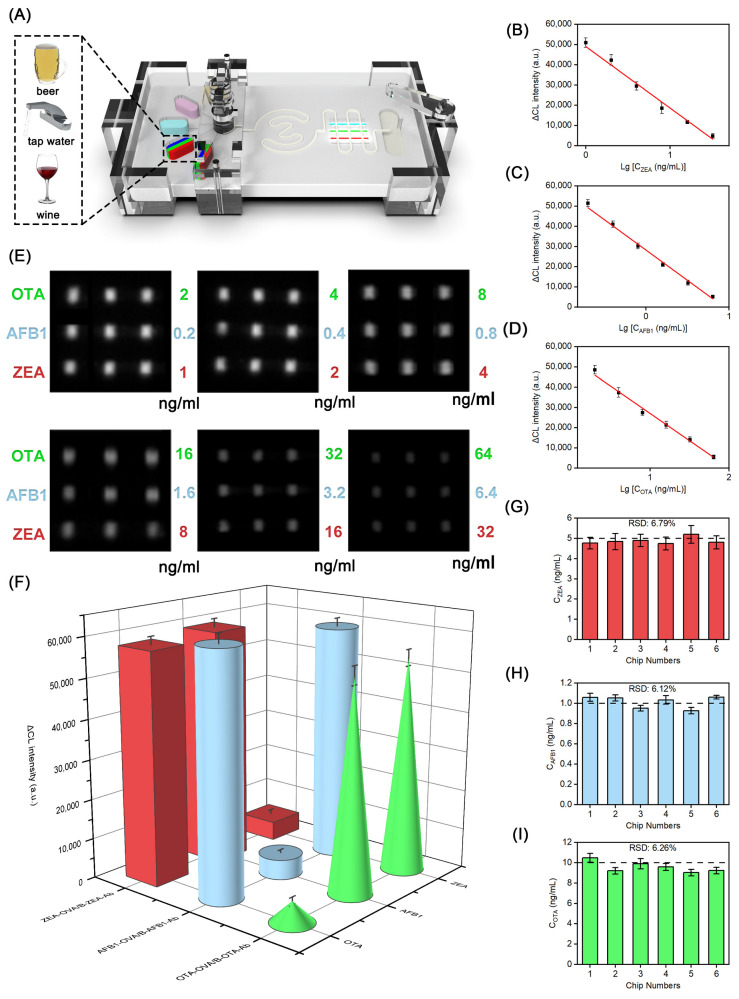
(**A**) Diagram of the microfluidic chip for real sample detection. Liner range of ZEA (**B**), AFB1 (**C**), and OTA (**D**). (**E**) CL intensity of ZEA, AFB1, and OTA in different combinations. (**F**) Specificity test of ZEA, AFB1, and OTA. Reproducibility of ZEA (**G**), AFB1 (**H**), and OTA (**I**).

We first tested a blank group of PBS-dissolved substances containing no mycotoxins by adopting optimized concentrations. According to the CL detected by CCD, the CL of the blank group is lower than the detection upper limit of the instrument. Then, we detected ZEA in the linear range of 1–32 ng/mL (Y = −30,433.957X + 49,022.968; R^2^ = 0.987) (Figure 7B), AFB1 in the linear range of 0.2–6.4 ng/mL (Y = −29,995.271X + 28,232.099; R^2^ = 0.993) (Figure 7C), and OTA in the linear range of 2–64 ng/mL (Y = −26,959.398X + 53,979.196; R^2^ = 0.992) (Figure 7D). The linear ranges cover the cut-off values of the three biomarkers. Y is the signal value of CL, and X is the logarithm of the mycotoxin concentration. The LODs of ZEA, AFB1, and OTA are 0.7619 ng/mL, 0.1374 ng/mL, and 0.9183 ng/mL according to the formula of three times signal-to-noise ratio. Since the LODs are well below the cut-off values, the sensitivity of these three biomarkers can meet the requirements. No cross-reactivity is a prerequisite for multiple detection of three biomarkers. We tested all ZEA, AFB1, and OTA combinations to see if they could make a significant difference. All other experimental procedures were the same. The ZEA, AFB1, and OTA concentrations were 32 ng/mL, 6.4 ng/mL, and 64 ng/mL, respectively. The experimental results showed that only combining specific antibodies and biomarkers would significantly reduce the CL intensity. In other cases, there was no significant change in the CL intensity (Figure 7), indicating that the method can detect multiple biomarkers. Repeatability is also an important index to evaluate the detection ability of this method. We used six chips from the same batch to detect the same biomarker. The ZEA, AFB1, and OTA concentrations were 5 ng/mL,1 ng/mL, and 10 ng/mL, respectively. The results showed that the coefficients of variation (CV) of ZEA (Figure 7G), AFB1 (Figure 7H), and OTA (Figure 7I) were 6.79%, 6.12%, and 6.26%, respectively, and CVs were all less than 10%, indicating that the method had good repeatability.

In these optimal conditions, the method was applied to detect ZEA, AFB1, and OTA concentrations in dry red wine samples. The results are shown in Table 1. By adding 1.25–20 ng/mL ZEA into wine samples, the recovery of ZEA was measured at 102.16 to 109.14% with relative standard deviations (RSDs) in the range of 7.18 to 12.10%. Similarly, we measured the recovery of AFB1 at 91.39 to 103.32%, the rate of RSD change at 6.21 to 8.31%, the recovery of OTA at 92.20 to 104.79%, and the rate of RSD change at 7.28 to 10.15%. Compared with other methods of detecting mycotoxins, SA-B Indirect competitive immunoassay also exhibited satisfactory performance in LOD, RSD, and recovery (Table 2). These results show that this method is reliable in real samples.

## 4. Conclusions

In summary, we constructed a PAMP integrated with a CL imaging system to realize the POCT of three mycotoxins in wine samples: ZEA, AFB1, and OVA. Based on the indirect immunoassay and SA-B signal system, the proposed PAMP can analyze the targets quantitatively. The prepared PAMP then demonstrated considerable specificity and sensitivity to three mycotoxins, including ZEA ranging from 1 to 32 ng/mL, AFB1 ranging from 0.2 to 6.4 ng/mL, and OTA ranging from 2 to 64 ng/mL. Compared with other methods, PAMP based on immune methods can avoid food background color and environmental interference. In addition, in real samples, PAMP demonstrated excellent recovery for ZEA, AFB1, and OTA. These findings confirm the practical applicability of our RCPW in real-world scenarios.

## Figures and Tables

**Table 1:** Mark recovery of ZEA, AFB1, and OTA in dry red wine samples.

ZEA			AFB1			OTA		
Spiked Value (ng/mL)	Recovery (%)	RSD (%)	Spiked Value (ng/mL)	Recovery (%)	RSD (%)	Spiked Value (ng/mL)	Recovery (%)	RSD (%)
1.25	106.76	7.18	0.3	103.32	6.21	3	103.45	10.15
2.5	105.35	10.22	0.6	95.56	7.72	6	104.79	7.28
5	109.14	12.10	1.2	96.86	8.07	9	96.46	9.08
10	102.16	9.91	2.4	91.39	6.99	18	92.20	9.66
20	105.09	9.52	4.8	97.75	8.31	36	95.82	8.59

**Table 2:** Comparison of the limit of detection (LOD), real sample analysis, and recovery rates between the SA-B indirect competitive immunoassay described in this article and other existing methods for detecting mycotoxins.

Methods	Colorimetric	Fluorescence	SERS	UPLC-Q-TOF/MS	Fluorescence	Chemiluminescence
Mycotoxins	OTA	OTA	ZEA	AFB1	ZEA AFB1 OTA	ZEA AFB1 OTA
LOD	2 μg/kg	0.17 ng/mL	3 μg/kg	0.2 μg/kg	400 ng/mL 5 ng/mL 40 ng/mL	0.7619 ng/mL 0.1374 ng/mL 0.9183 ng/mL
Real samples	grape juice	coffee, wine	corn	chili powder	corn	dry red wine
Recovery (%)	99.4–104.2	99.2–100.9	90.6–105.3	78.8–100.8	72.9–105.8%	91.4–109.1
RSD (%)	<2%	1.45–4.67	2.56–7.62	1.6–7.7	-	6.21–12.10
References	[47]	[48]	[49]	[50]	[51]	This work

## Data Availability

The original contributions presented in the study are included in the article, further inquiries can be directed to the corresponding author.
